# A Novel Surgical Approach to Synovial Sarcoma of the Esophagus: New Possibilities for the Management of a Rare Tumor

**DOI:** 10.7759/cureus.68211

**Published:** 2024-08-30

**Authors:** Luke W Schultz, Kevin W Chen, Corbin D Sullivan

**Affiliations:** 1 Department of Otolaryngology, Head and Neck Surgery, Western Michigan University Homer Stryker M.D. School of Medicine, Kalamazoo, USA; 2 Department of Surgery, Western Michigan University Homer Stryker M.D. School of Medicine, Kalamazoo, USA

**Keywords:** esophageal tumor, case report, esophagectomy, esophagoscopy, esophageal synovial sarcoma

## Abstract

Synovial sarcoma (SS) is an uncommon soft tissue malignancy that typically occurs in surrounding joints. Rarely, this malignancy may occur in the gastrointestinal (GI) tract, including the esophagus. Given the rarity of this malignancy, treatment recommendations are limited. In all reviewed cases, surgical resection was performed via some variation of esophagectomy. Here, we present a novel approach to the treatment of esophageal SS.

A 30-year-old patient was found to have a large upper esophageal mass after developing symptoms of dysphagia, cough, and regurgitation. Otolaryngology was consulted for the removal of the mass. A CO_2_ laser was used to remove the pedicled mass from the esophageal wall. Given the size of the mass, it could not be removed through the upper esophageal sphincter despite significant effort. The decision was made to allow the mass to pass to the stomach to be digested. The patient had an uncomplicated postoperative course with seemingly complete spontaneous digestion of the tumor and no evidence of residual tumor, metastasis, or recurrence. Frozen section pathology confirmed SS of the esophagus.

SS of the esophagus is a rare malignancy that presents unique treatment challenges. This case represents a novel strategy for resecting esophageal SS utilizing transoral esophagoscopy. The preservation of the upper esophageal sphincter may also be accomplished using this approach by allowing for the autodigestion of tumors in some cases. This case also suggests that esophagectomy may not be necessary for all cases of esophageal SS.

## Introduction

Synovial sarcoma (SS) is an uncommon malignancy that most commonly develops in children and young adults. This rare malignancy is named for its histologic resemblance to the synovium. It accounts for less than 10% of all soft tissue sarcomas [1]. This malignancy typically develops in soft tissue areas surrounding the joints, with the extremities being the primary site of disease in most cases [2]. Rarely, the primary tumor may develop in the GI tract. This atypical presentation has been reportedly misdiagnosed as a fibrovascular polyp, sarcomatoid carcinoma, or gastrointestinal stromal tumor [3]. Here, we present a case of primary synovial sarcoma of the esophagus, which we believe to be the sixteenth reported case [3,4]. Currently, there is limited data to guide treatment of this rare malignancy. The current mainstay of treatment is surgical resection with adequate margins when feasible with an unclear role for adjuvant radiotherapy or chemotherapy [5,6]. The presence of residual tumor or unsure margins support the use of adjuvant agents while some view deferral of adjuvant treatment as reasonable with adequate resection [1]. While the specifics of past resections of esophageal SS are dependent on tumor location, all available reports include some variation of esophagectomy with the addition of chemo/radiation therapy in most cases [1,5-9]. Given the invasiveness, complication rate, and challenging recovery of esophagectomy, alternative surgical approaches would be appealing from a quality-of-life standpoint.

## Case presentation

**Figure 1:**
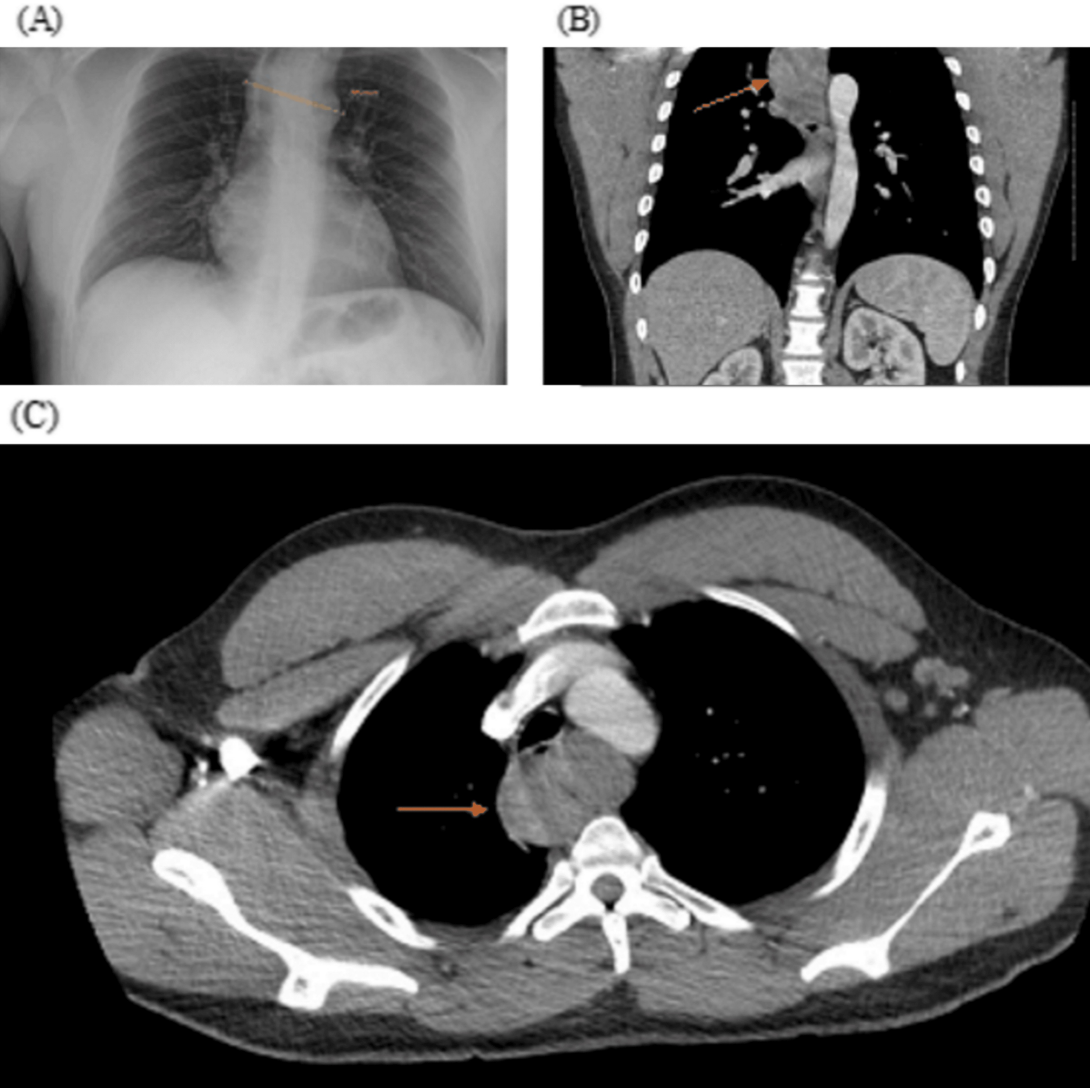
Preoperative imagining (A) Upright chest X-ray with the mediastinal mass measuring 85 mm. (B) Coronal and (C) Axial CT scan demonstrating esophageal soft tissue mass (arrows indicate the mass)

We present the case of a 30-year-old male with a history of schizophrenia and seizure disorder who initially presented to the emergency department for evaluation of dysphagia, cough, and regurgitation following meals. A mediastinal mass was identified on chest X-ray, with a subsequent computed tomography (CT) scan showing an 8 x 5 x 3.2 cm upper thoracic esophageal mass causing anterior displacement of the intrathoracic trachea (Figure 1). The patient was admitted and underwent esophagogastroduodenoscopy (EGD) with biopsy. The initial biopsy returned non-diagnostic with necrotic material present. Repeat EGD/biopsy revealed ulcerated granulation tissue and necrotic debris without evidence of malignancy. Given its endoscopic appearance, a fibrovascular polyp of the cricopharyngeus with esophageal prolapse was suspected. At this point, otolaryngology was consulted, and the patient was discharged with the planned removal of the mass in the outpatient setting.

During the operation, the esophagoscope was first used to identify the mass, which was then biopsied and sent for a frozen section. Pathologic analysis initially indicated a spindle cell neoplasm. The mass was isolated using a bivalve upper esophagoscope and was seen to be pedunculated at the left superior esophageal wall. A CO_2_ laser set to 70 mJ 3-watt ultrapulse was used to cut across the pedicle. Frozen sections were sent, indicating negative margins. Hemostasis was achieved using suction cautery. The mass was attempted to be removed through the upper esophageal sphincter in all orientations. Eventually, after considerable time and effort, it was determined that the tumor was too large to be extracted through the upper esophageal sphincter, and the decision was made to allow it to pass through the stomach to be digested. A nasogastric (NG) tube was placed, and the patient was placed on a clear liquid diet. A post-procedure esophagram was completed the following day without evidence of a leak. The patient tolerated oral intake well and was discharged on postoperative day two. Upon second opinion pathology referral to an outside facility, diagnosis of biphasic synovial sarcoma was favored. Immunohistochemistry demonstrated immunoreactivity for TLE, SS18-SSX, SSX-C-term, CK Oscar, EMA, CD99, and Bcl-2.

Following his operation, the patient followed up with radiation oncology at an outside facility, at which point they decided to proceed with active observation and consideration of radiation/chemotherapy should there be evidence of incomplete resection, metastasis, or recurrence. Over the 11 months since his operation, the patient underwent CT chest (3), whole-body positron emission tomography (PET), CT chest/abdomen/pelvis, and EGD. All were negative. The patient has no complaints postoperatively and notes complete resolution of dysphagia, cough, and vomiting.

## Discussion

This case represents a novel approach to an extremely rare tumor. To our knowledge, this is the sixteenth reported primary SS of the esophagus ​[3,4]​. While rare, SS should be considered in cases of an esophageal mass, particularly those of polypoid appearance and those with an initially inconclusive biopsy. These tumors tend to present in the upper third of the thoracic esophagus in patients of variable age [5]. The differential diagnosis includes gastrointestinal stromal tumor, fibrovascular polyp, polypoid carcinoma, and inflammatory polyp, with definitive diagnosis often requiring histologic evaluation. Immunohistochemistry is often immunoreactive for TLE1+, EMA+, BCL2+, CD99+, with characteristic translocations involving SS18 and SSX1, SSX2, or SSX4 [5]. While esophageal SS is typically treated with esophagectomy, this appears to be the first to be resected via transoral esophagoscopy. This is also the first known case of a tumor being left to be digested in the patient’s GI tract, thus preserving the upper esophageal sphincter and hastening recovery ​[6]. This practice may be feasible in select situations, though further study is needed to draw definitive conclusions. This case suggests that esophagectomy, with associated significant recovery time, functional deficits, and relatively high complication rate, may not be necessary for all cases of esophageal SS ​[10,11]. The role of conservative surgical management of SS in other soft tissue sites, including the larynx does have support in the literature, assuming clear margins can be attained [12]. Although surgical intervention remains the mainstay of treatment, esophageal malignancy is often also treated with adjuvant therapy ​[13]. However, decisions regarding the utilization of adjuvant therapy are difficult due to the limited data relating to the management of this rare entity. This case preliminarily supports surgical intervention followed by active observation over adjuvant chemo/radiation therapy for the management of esophageal SS thus avoiding unnecessary treatment.

## Conclusions

Despite its rarity, synovial sarcoma of the esophagus should be considered during workup for esophageal mass. The esophagoscopic approach may be reasonable for resection of select tumors, which may negate the need for more invasive procedures such as esophagectomy. Drawing conclusions regarding the utilization of adjuvant radiation therapy versus active observation will require more patients with longer follow-ups. In this case, allowing tumor autodigestion eased recovery and maximized patient quality of life, though the utility of this practice is unclear.
